# Effect of different finishing and polishing systems on surface properties of universal single shade resin-based composites

**DOI:** 10.1186/s12903-024-03958-8

**Published:** 2024-02-07

**Authors:** Ghada Alharbi, Hend NA Al Nahedh, Loulwa M. Al-Saud, Nourah Shono, Ahmed Maawadh

**Affiliations:** 1 Department of Restorative Dental Science, College of Dentistry, King Saud University, P.O. Box 60169, Riyadh, 11545 Saudi Arabia; 2 Department of Clinical Dental Sciences, College of Dentistry, Princess Nourah bint Abdulrahman University, Box 84428, Riyadh, PO 11671 Saudi Arabia

**Keywords:** Universal single-shade RBCs, Surface roughness, Surface gloss, Vickers microhardness

## Abstract

**Background:**

Recently, universal single-shade resin composites have become increasingly available in the dental market. The modification of their composition can have an inadvertent effect on their physical and surface properties, and subsequently determinantal effect on their clinical function and longevity. Therefore, this study aimed to evaluate the effect of different finishing and polishing (F/P) systems on surface roughness (Ra), surface gloss (GU), and Vickers microhardness (VMH) of universal single-shade RBCs.

**Materials and methods:**

Four commercial RBCs were used; the universal single-shade RBCs were Omnichroma, Charisma® Diamond ONE, and Vittra APS Unique, and a conventional nanocomposite Filtek™ Z250 XT was used as a control. The 3 F/P systems were Sof-Lex™ XT, Enhance®/PoGo®, and Diacomp® Plus Twist. A total of 160 discs were used for the 3 F/P system groups for all RBCs (*n* = 10). After F/P, the Ra, GU, and VMH were assessed. The data were analyzed using 2-way ANOVA at *p*-value < 0.05.

**Results:**

Significant differences were found among the four RBCs and the 3 F/P systems (*p* < .000). Omnichroma showed the lowest Ra and acceptable GU, but the lowest VMH. Charisma showed the highest Ra, acceptable GU, and VMH. Vittra showed acceptable Ra, GU, and VMH and Filtek showed the highest GU, VMH, and acceptable Ra.

**Conclusion:**

Although conventional nanohybrid RBC (Filtek Z250 XT) showed better GU and VMH values, the universal single-shade RBCs demonstrated comparable surface properties. The highest GU & VMH and lowest Ra were achieved by Diacomp followed by Enhance and Soflex.

## Introduction

Resin-based composites (RBCs) have become one of the most widely used direct restorative materials in dentistry due to their good esthetic and mechanical properties, versatility, conservative approach, reparability, and good clinical performance [1–5]. Understanding the different properties of RBCs before clinical use will help in selecting the optimum restoration, which will in turn lead to ideal outcomes in the intraoral environment [6].

The surface properties of restorative materials such as surface roughness, gloss, and hardness should be considered to provide the esthetic and functional requirements of the tooth being restored [7]. Surface roughness refers to the finer irregularities of the surface texture that usually result from the production process or the material’s characteristics [8]. Composite surface roughness affects the amount of plaque accumulation on the surfaces of RBCs and thus influences the durability and esthetic appearance of the restorative material [9, 10]. Surface treatment with an appropriate finishing and polishing technique is considered a critical procedure to achieve a favorable esthetic result and increase the longevity of the dental restoration [11].

Surface gloss is defined as the ability of the material to reflect direct light [12]. The gloss of RBC is influenced by surface roughness, the light reflected from each angle of the surface, and the particle size of the material, which is related to the amount of light reflected from the material itself [13]. Therefore, a smoother surface has a higher gloss, indicating better clinical durability and better esthetic appearance [14].

Surface hardness is a property of the material which is important for maintaining the stability of restorations, it refers to the resistance of the material to indentation [15]. Most researchers choose the Vickers and Knoop hardness tests to investigate the hardness of dental materials [16]. Hardness is one of the most important properties of RBCs and it is related to compressive strength, wear resistance, and degree of conversion [17]. Furthermore, a low hardness value of a resin composite indicates poor chemical/physical bonding between the matrix and the filler interface [18].

To obtain the desired esthetics and at the same time ensure the longevity of the restorations, the finishing and polishing ability of the RBCs should be optimized [8]. Finishing removes any scratches caused by contouring instruments and results in a smooth surface. Polishing is the final step that reduces the surface roughness and provides the restoration with an enamel-like glossy surface [19]. A rough surface or a sub-optimally finished and polished restoration can have a major influence on staining, plaque retention, gingival inflammation, and secondary caries, and may lead to restoration failure [20, 21]. Therefore, optimizing the finishing and polishing procedures would decrease the surface roughness, achieve a high gloss level, and consequently improve the surface hardness of the RBCs [22, 23].

Color matching of the RBCs to the tooth structure has been a considerable challenge for a long time [24]. The availability of different shades and technique sensitivities render shade selection a very complicated process [25]. The term blending effect (color assimilation or induction) describes the ability of a material to assume a similar color to the surrounding tooth structure [26]. The simplification process of color matching started with the group-shade composites until the production of a universal single-shade composite material that claims to match different tooth shades [27, 28]. The concept of single-shade RBCs was introduced to describe resin composites designed to esthetically simulate all shades with only one nominal shade [26]. These materials are produced to perfectly match the surrounding tooth color, regardless of the color of the tooth to be restored since they have the ability to combine and acquire a color similar to that of its surrounding structures [27].

Examples of universal single-shade RBCs are Omnichroma (Tokuyama Dental, Tokyo, Japan) [29], Charisma® Diamond ONE (Kulzer GmbH, Hanau, Germany) [30], and Vittra APS Unique (FGM, Joinville, SC, Brazil) [31] which have recently been introduced into the market. Omnichroma is the first single-shade universal RBC that was released in 2019, according to the manufacturer (Tokuyama Dental), it is a supra nanofillled RBC that features innovative technological approaches and contains no pigments. Its optical properties are based on structural color, a “smart chromatic technology” in which the RBC responds to light waves of a specific frequency by perfectly reflecting that specific wavelength within the tooth color space [26]. Charisma Diamond One (Kulzer) is another single-shade universal RBC, which is a nanohybrid composite. It is based on the concept of “adaptive light matching,” in which the restoration shade is achieved by absorbing the wavelengths reflected from the surrounding tooth shade [32]. Vittra APS Unique (FGM) is also a single-shade RBC, which is a nanohybrid composite. It has a blending effect that copies the shade of the tooth substrate during the polymerization process. In addition, the manufacturer claims that the exclusive Advanced Polymerization System (APS) technology, which consists of a combination of different photoinitiators that interact among themselves amplifies the curing capacity of the light emitted by the light-curing unit. APS provides high polymerization strength, which allows for a higher conversion rate and better mechanical properties [33].

Several studies reported positive color-matching results of universal single-shade composites [26, 32, 34–36]. A recent study evaluated the color matching between single-shade composite resins and multi-shade composite resins [37]. They found that all groups demonstrated acceptable color matching; however, single-shade composite resins showed better matching values than multi-shade resins. They emphasized that single-shade composite resins simplify the shade-selection process and are promising materials for use in the dental practice [37]. Another recent study evaluated the visual (CAP-V) and instrumental (CAP-I) color-matching potential for three single-shade composite resins (Omnichroma, Charisma Diamond One, Vittra APS Unique) [38]. They found that single-shade resin composites have acceptable CAP and the use of single-shade resin composites can reduce in-chair clinical times by minimizing the time spent on the shade selection [38].

The modification of single-shade RBCs composition can have an inadvertent effect on their physical and surface properties and subsequently determinantal effect on their clinical function and longevity. Therefore, evaluation of these properties of the newly introduced universal single-shade RBCs is imperative.

The purpose of this research was to evaluate the effect of different finishing and polishing systems on surface roughness, surface gloss, and Vickers microhardness of universal single-shade RBCs and compare them with a conventional nanocomposite restorative material. The null hypotheses were: 1- There would be no statistically significant difference between the different F/P systems on the Ra of the universal single-shade RBCs and with a conventional nanocomposite. 2- There would be no statistically significant difference between the different F/P systems on the GU of the universal single-shade RBCs and with a conventional nanocomposite. 3- There would be no statistically significant difference between the different F/P systems on VMH of the universal single-shade RBCs and with a conventional nanocomposite. 4- There would be no statistically significant correlation between the Ra, GU & VMH of the universal single-shade RBCs and with a conventional nanocomposite.

## Materials and methods

**Fig. 1:**
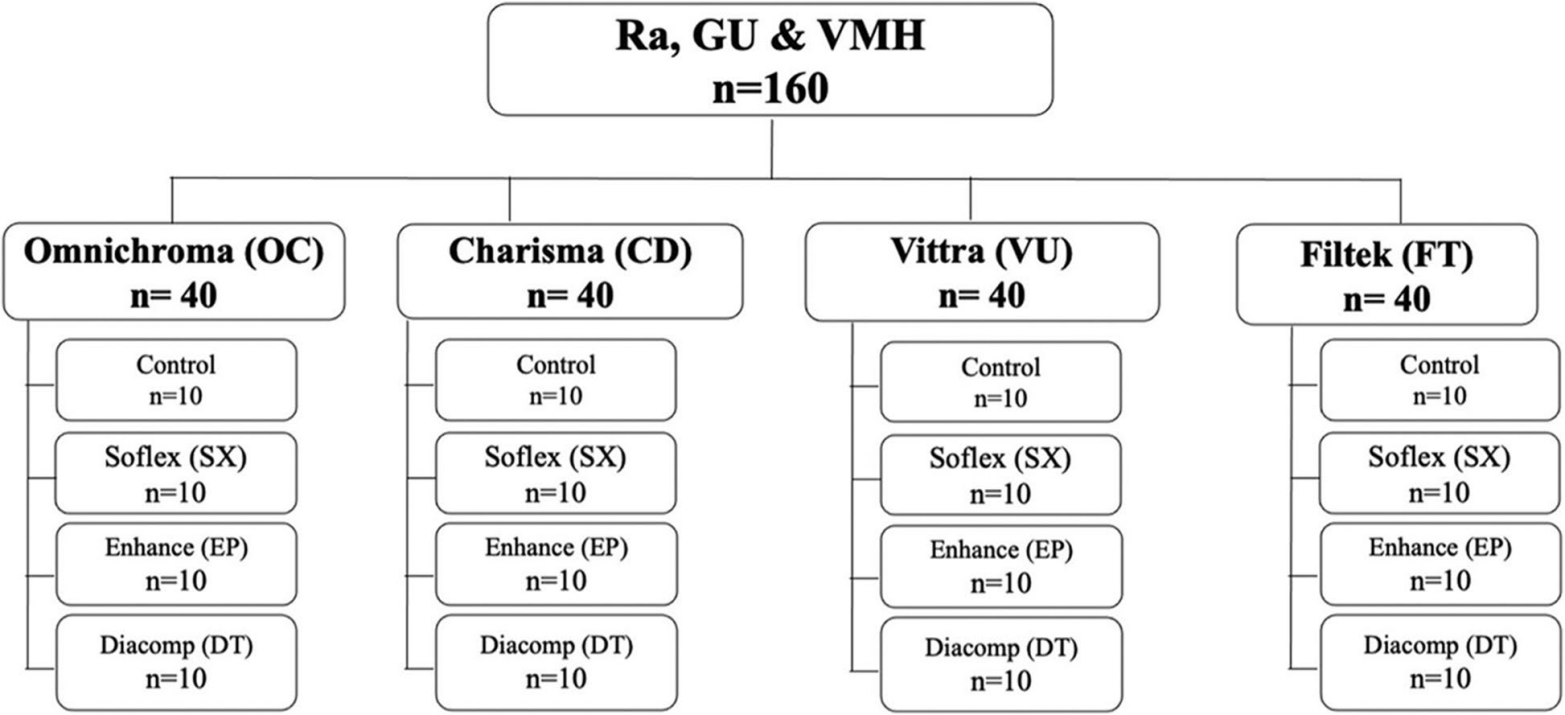
The experimental groups in this study. Ra = Surface Roughness, GU = Surface Gloss, VMH = Vickers Microhardness

Four commercial resin composites and three finishing and polishing systems were used in this study as shown in Tables 1 & 2. The universal single shade RBCs are Omnichroma (Omnichroma/OC), Charisma® Diamond ONE (Charisma/CD), and Vittra APS Unique (Vittra/VU), and the conventional RBC is Filtek™ Z250 XT (Filtek/FT) (3 M ESPE, St. Paul, MN, US), was used as a control. The three finishing and polishing (F/P) systems are Sof-Lex™ XT (Soflex/SX) (3 M ESPE, St. Paul, MN, US), Enhance®/PoGo® (Enhance/EP) (Dentsply, Milford, DE, US), and Diacomp® Plus Twist (Diacomp/DT) (EVE Ernst Vetter GmbH; Pforzheim, Germany). Ten specimens of each RBC were prepared. Thus, a total of 160 discs were used for the three properties that were measured (Fig. 1).

**Table 1:** List of resin-based composites (RBCs) used in this study

Material	Composition	Filler Type (wt/vol)	Lot.no	Manufacturer
**OMNICHROMA** (OC) (One Shade)	**Filler:** Uniform sized supra-nano spherical filler (SiO2-ZrO2 260 nm), round-shaped composite filler (containing 260 nm spherical SiO2-ZrO2). **Base resin:** UDMA, TEGDMA	Supra-Nanofilled. (79 wt%, 68 vol%)	(030E81)	Tokuyama Dental, Tokyo, Japan
**Charisma® Diamond ONE** (CD) (One Shade)	**Filler:** Barium Aluminium Boro Fluor Silicate Glass (contains approximately 64% filer by volume, its filler particle size is 5 nm-20 μm). **Base resin:** TCD-Urethaneacrylate, Silica, UDMA, TEGDMA. Titanium Dioxid, Fluorescent Pigments, Metalic Oxide Pigments, Organic Pigments, Aminobenzoic-acid-ester, BHT, Camphorquinone. Bisphenol A (BPA) free products	Nanohybrid (81 wt%64 vol%)	(K010022	Kulzer GmbH, Hanau, Germany
**Vittra APS UNIQUE** (VU) (One Shade)	**Filler:** Boron-aluminum-silicate glass. **Base resin:** A mixture of methacrylate monomers, Photoinitiator with advanced polymerization system (APS), co-initiators, stabilizers, and silane. Bisphenol A (BPA) free products	Nanohybrid (72–80 wt%, 52–60 vol%)	(230921)	FGM Joinville, SC Brazil
**Filtek™ Z250 XT** (FT) (Shade A2)	**Filler:** Silica particle 20 nm and Zirconia/Silica particle. **Base resin:** BIS-GMA, UDMA, BIS-EMA, TEGDMA &PEGDMA	Nanohybrid (82 wt% 68 vol%)	(NE58072)	3 M Dental Products ESPE, St. Paul, Minnesota, United States

*Bis-GMA* Bisphenol A diglycidildimethacrylate: *UDMA* Urethane dimethacrylate: *TEGDMA* Triethylene glycol dimethacrylate*: Bis-EMA* Bisphenol A ethoxylated dimethacrylate: *PEGDMA* Polyethylene glycol dimethacrylate: *HEMA* 2-hydroxyethyl methacrylate: *TCD* tricyclodecane: *BHT* butylhydroxytoluene: *Omnichroma/OC* Omnichroma Charisma: *CD* Charisma® Diamond ONE: *Vittra/VU* Vittra APS Unique: *Filtek/FT* Filtek™ Z250 XT.

**Table 2:** List of finishing and polishing (F/P) systems used in this study

Material	No. of Steps	Types	Composition	Lot.no	Manufacturer
Sof-Lex™ XT Discs (SX)	3 steps	Medium disk (40 μm-dark orang) Fine disk (24 μm-light orange) Superfine disk (8 μm-yellow)	Aluminum oxide coated disc	(030E81)	Tokuyama Dental, Tokyo, Japan
Enhance®/ PoGo® (EP)	2 steps	Finisher disk aluminum oxide (40 μm- white) Diamond-coated micro-polisher (10–15 μm- grey)	Polymerized Urethane Dimethacrylate Resin; Aluminum Oxide; Silicon Dioxide, Fine Diamond Powder	(K010022)	Kulzer GmbH, Hanau, Germany
DIACOMP® PLUS TWIST (DT)	2 steps	Medium pre-polisher (pink) Fine high-shine polisher (grey)	Diamond impregnated rubber spiral wheel	(230921)	FGM Joinville, SC Brazil

*C* Control (No finishing & polishing): *Soflex/SX* Sof-Lex™ XT: *Enhance/EP* Enhance/® PoGo®: *Diacomp/DT* Diacomp® Plus Twist.

### Samples size determination

Sample size determination was performed using G Power v3.1.9.4 software (Düsseldorf, Germany) to determine the minimum sample size required to test the study hypotheses. Results indicated the required sample size to achieve 85% power for detecting a medium effect according to Cohen’s guidelines [39] at a significance criterion of α = 0.05 was *N* = 160 (10 per group) for the two-way ANOVA test. Thus, the obtained sample size of *N* = 160 is adequate to test the study hypotheses.

### Samples preparation

Forty specimens of each RBC were prepared for all F/P groups (n = 10). A total of 160 specimens were used for surface roughness, surface gloss, and Vickers microhardness tests. The specimens were fabricated using a split customized stainless-steel mold (10 mm diameter × 2.0 mm height). After each composite was placed, the mold was compressed between two glass microscope slides using finger pressure to remove excess material and obtain a flat surface. All of the samples were polymerized according to manufacturer instructions through a 1 mm thick glass slide and polyester matrix using a Bluephase N LCU (Ivoclar Vivadent, Schaan, Liechtenstein, Switzerland) at an intensity output of (1200 mW/cm^2^) for 20 s, the power intensity was measured using a dental Bluephase radiometer (Ivoclar Vivadent, Schaan, Liechtenstein, Switzerland). The composite specimens were removed from the molds after light-curing. For complete polymerization, all specimens were stored in distilled water at 37 °C in an incubator for 24 hours before testing (JSGI-150 T, Korea). The top surfaces of the specimens were ground with 800-grit silicon carbide (SiC) paper for 20 s under stream water. Samples were embedded in an ortho resin mold (Interacryl Ortho, Interdent, Slovenia) allowing the top surface to be exposed for testing. The embedded samples were secured in a custom-made holder with a base allowing the sample to be oriented parallel to the floor, and a vertical holder allowing multi-orientation for the handpiece and horizontal movement for the finishing and polishing systems in sweeping forward and backward motions where the finishing instrument is held flat in firm contact with the surface of the composite without excessive pressure. One operator was trained to perform the finishing procedures consistently for all the samples and new finishing and polishing discs were used according to the manufacturer’s instructions.

### Finishing and polishing

To control the variability, one operator was blinded to the composite material type being processed, and all the finishing and polishing procedures were performed in a randomized order. Each F/P procedure was carried out using the same low-speed handpiece (NSK, Tokyo, Japan; average 10,000 rpm) with light pressure and the application time was 60 s for each system. The groups were distributed as:

1. Group 1: Control (No finishing and polishing).
2. Group 2: Sof-Lex™ XT (SX; 3 M ESPE): Soflex discs were applied without water spray in three steps; medium (dark orange), fine (light orange), and superfine (yellow). The discs were applied for 20 s for each step, followed by rinsing, and drying with an air/water syringe for a total of 10 s after each step.
3. Group 3: Enhance®/PoGo® (EP; Dentsply); Enhance was applied without water spray in two steps; the surface was treated with Enhance (white) followed by Pogo (grey) under light pressure for 30 s for each step; followed by rinsing and drying with an air/water syringe for a total of 10 s after each step.
4. Group 4: Diacomp® Plus Twist.; Diacomp as applied without water spray in two steps; the surface was treated with a pre-polisher medium (pink) rubber spiral wheel followed by a high shine polisher fine (grey) rubber spiral wheel under light pressure for 30 s for each step; rinsed and dried with a water/air syringe for a total of 10 s after each step.

### Surface roughness measurement

For the surface roughness, 10 specimens were measured for each RBC and F/P system. Characterization and imaging were performed using a Contour GT-K 3D Optical Microscope (Contour GT-K 3D Optical Microscope Bruker®, Tucson, AZ, USA). 3D non-contact surface metrology was determined with interferometry. Samples were measured using vertical scanning interferometry which uses a broadband (normally white) light source which is effective for measuring objects with rough surfaces, as well as those with adjacent pixel-height differences greater than 135 nm. Each sample was scanned at 3 equidistant positions at 3 intervals and averaged accordingly to determine the roughness (Ra) value.

### Scanning Electron microscopy observation

Three specimens from each composite and F/P system were selected (highest, median, and lowest value of Ra), and scanning electron microscope (SEM) images were obtained. Each specimen was analyzed qualitatively using SEM (JEOL JSM-6360LV, JEOL, Tokyo, Japan) after sputter coating with gold (JEOL ION SPUTTER JFC-1100, JEOL, Tokyo, Japan) for 5 min with 10 mA current with an accelerating voltage of 15 kV. The surfaces were examined at × 50 to × 500 and representative photomicrographs were taken for each sample.

### Surface gloss measurements

For the surface gloss, 10 specimens were used for each RBC and F/P system. Gloss measurements, which are expressed in gloss units (GU), were performed using a gloss meter (Novo-Curve Gloss meter, East Sussex, UK) with the light source and detector both set at 60° to normal. Before measurement, the gloss meter was calibrated to a standard gloss board (Gs (60°) = 100.4). The gloss value of 100.4 obtained with a glass board was considered the reference value. Thus, the shinier a surface was, the closer the value was to 100%. The instrument measures the intensity of a reflected light beam after striking the surface and compares the measured value to a reference value. An opaque black plastic mold was placed over the specimen during measurement to eliminate the influence of ambient light and maintain the exact position of the sample for repeated measurements. Three measurements were obtained for each specimen, and the average value was determined.

### Vickers microhardness measurement

For the microhardness tests, 10 specimens were used for each RBC and F/P system. The Vickers hardness number (VHN) was determined using Micro Vickers hardness testing machines (Innova Test, Nova 130/240 series, Netherlands). Three indentations were made in a triangular configuration on the center of the top surface only, under a 200 g load with a 15 s dwell time. The indentations were made at equal distances apart and 1 mm away from adjacent indentations and specimen margins. The average hardness values for each specimen were then calculated.

### Statistical Analysis

Data were analyzed using SPSS 26.0 Windows version statistical software for Windows (IBM, Inc., Chicago, USA). Descriptive statistics (mean and standard deviation) were used to describe the quantitative outcome variables. The results were first tested for normality using the Shapiro–Wilk test. The results of the effect of different finishing and polishing systems on surface roughness, surface gloss, and Vickers microhardness and their comparison with a conventional nanocomposite restorative material were carried out by using a two-way analysis of variance (4 × 4 ANOVA) followed by Tukey’s test for multiple comparisons. All tests were performed at a significance level of *p* ≤ 0.05. The non-parametric Spearman’s correlation coefficient was used to quantify the correlation between Ra, GU, and VMH for each tested material.

## Results

### Surface roughness

The mean values and standard deviations of surface roughness (Ra, μm) for each resin composite and F/P system are presented in Table 3. The results indicated statistically significant differences (*p* < .000) and interaction effect between resin composite type and F/P systems, F (9,144) =334.48, *p* = .000, partial Eta squared (*η*_p_^2^) = .453.

**Table 3:** The Mean (± SD) surface roughness values (Ra, μm) of the tested resin composites for each group of the finishing and polishing systems

Resin Composite		Finishing and Polishing System		
	Control	Soflex	Enhance	Diacomp
**Omnichroma**	1.409 ± 0.13 ^a^	0.475 ± 0.05 ^b^	0.306 ± 0.05 ^c^	0.129 ± 0.02 ^d^
**Charisma**	2.551 ± 0.26 ^a^	1.506 ± 0.17 ^b^	1.003 ± 0.09 ^c^	0.678 ± 0.09 ^d^
**Vittra**	2.055 ± 0.21^a^	0.742 ± 0.12^b^	0.332 ± 0.06 ^c^	0.145 ± 0.02 ^d^
**Filtek**	2.004 ± 0.26 ^a^	0.623 ± 0.08 ^b^	0.375 ± 0.05 ^c^	0.188 ± 0.02 ^d^

Different superscript letters indicate statistically significant differences between the different F/P systems for each composite material (*P* < .05).

**Fig. 2:**
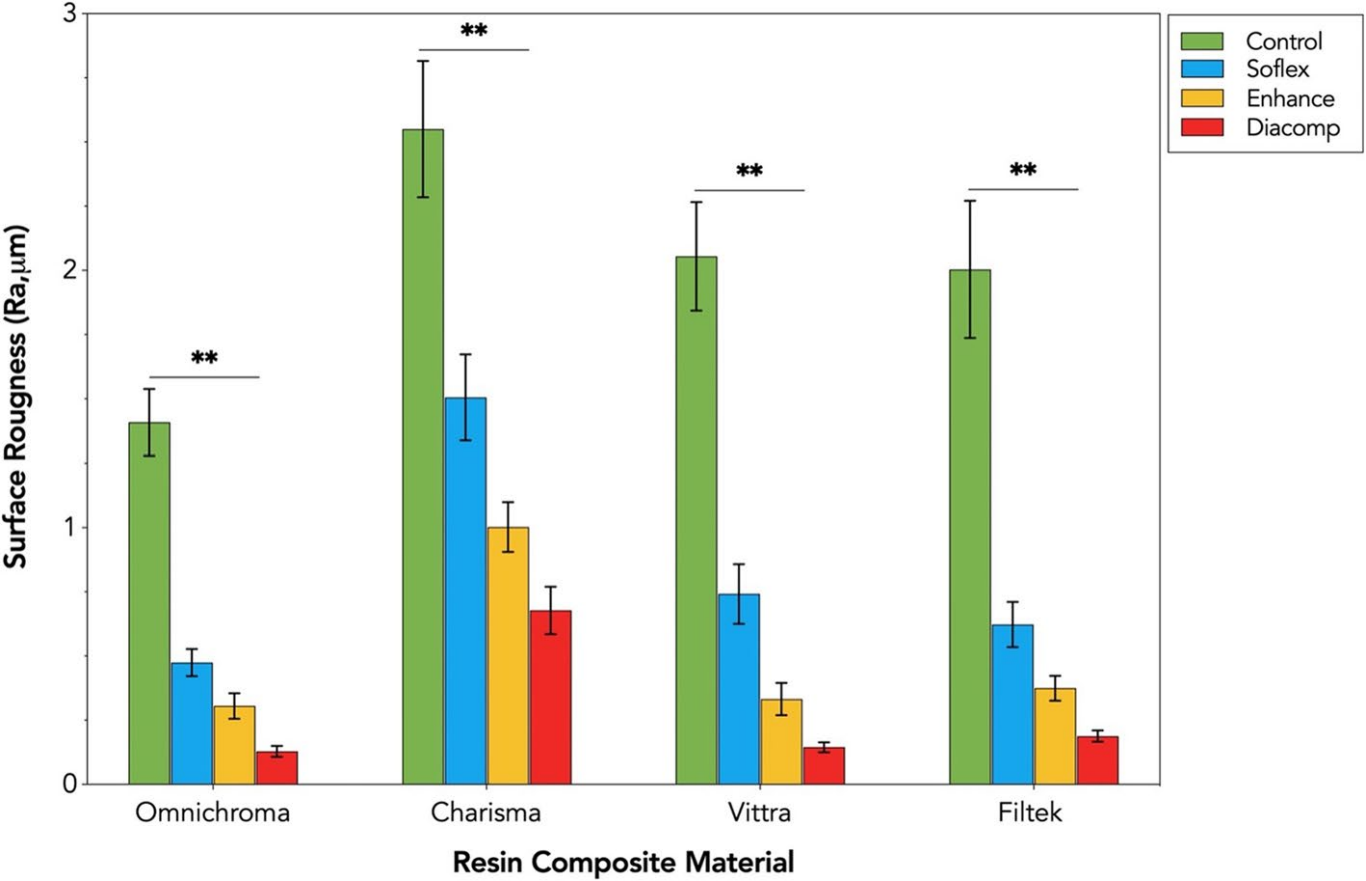
Mean surface roughness values (Ra, μm) of the tested resin composites based on the groups of finishing and polishing systems. Error bars represent ± SD. Double asterisks denote statistically significant differences at (*p* < .0001) among the different groups

Pairwise multiple comparisons with Tukey’s post hoc test showed Omnichroma has the smoothest surface with significantly lower Ra values than all other tested materials (*p* < .000) (Fig. 2). The least smooth surface was observed for Charisma, with significantly higher Ra values than all other tested materials (*p* < .000). No statistically significant difference was found between Vittra and Filtek (*p* = 0.894). The order of composites ranked from the highest to the lowest Ra in the Control (no finishing & polishing) & Soflex groups were Charisma < Vittra < Filtek < Omnichroma and in Enhance & Diacomp groups was Charisma < Filtek < Vittra < Omnichroma.

Regarding the F/P system, Tukey’s post hoc test showed that the use of Diacomp resulted in the smoothest surface of resin composites, with significantly lower Ra values than with any other tested systems (*p* < .000) (Fig. 2). The least smooth surface was observed after using Soflex, with significantly higher Ra values than all other tested systems (*p* < .000), but it was smoother compared only to the Control (no finishing & polishing) group. The order of the F/P system ranked from the highest to the lowest Ra was Control < Soflex < Enhance < Diacomp.

### SEM observations

**Fig. 3:**
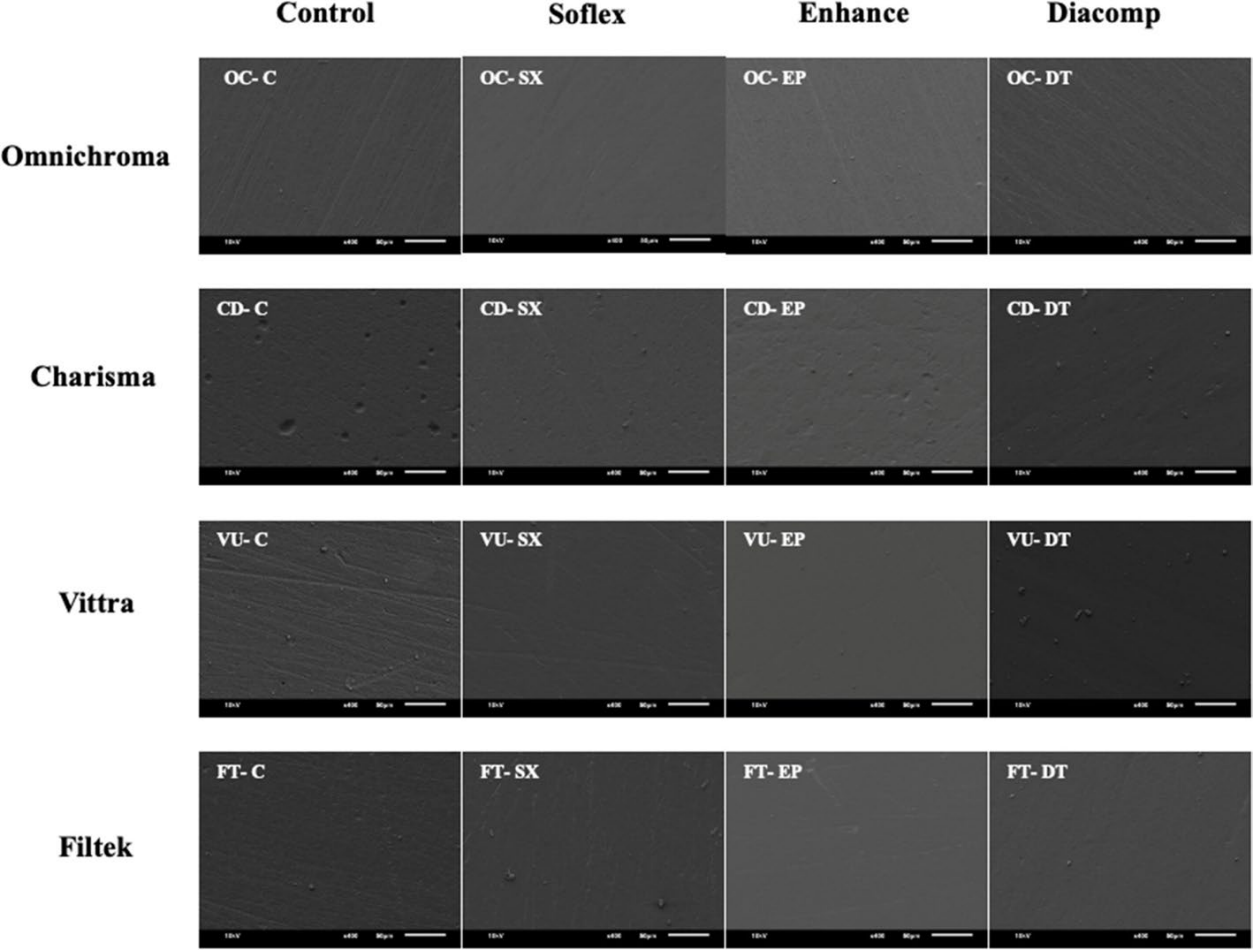
Representative SEM images of the resin-based composite surfaces after different finishing and polishing methods

SEM images of the resin-based composite surfaces after different finishing and polishing methods are depicted in Fig. 3. Generally, Different surface topographies were observed for the different combinations of composite materials and F/P systems. Omnichroma has the smoothest surface with a lower number of depth and scratches compared to other tested materials. The roughest surface was observed for Charisma, with multiple scratches. The rough surface of Charisma appears to be more due to pitting in the surface of the control specimens, while the finished and polished specimens are related to the pits and small protrusions from the surface. Vittra and Filtek showed very similar patterns with regard to surface appearance. Also, SEM images showed that using Diacomp resulted in the smoothest surface of resin composites compared to other tested materials. The least smooth surface was observed after using Soflex, but it was smoother than the Control (no finishing & polishing) group.

### Surface gloss

The mean gloss values (GU) and standard deviations for each resin composite are presented in Table 4. The results indicated statistically significant differences (*p* < .000) and interaction effect between resin composite type and F/P systems, F (9,144) = 233.53, *p* = .000, partial Eta squared (*η*_p_^2^) = .326.

**Table 4:** Average gloss values (GU) and standard deviations ( ± SD) of the tested resin composites for each group of the finishing and polishing systems

Resin Composite		Finishing and Polishing System		
	Control	Soflex	Enhance	Diacomp
**Omnichroma**	33.79 ± 1.64^a^	51.25 ± 2.34^b^	55.73 ± 3.79^c^	57.26 ± 2.87^c^
**Charisma**	21.2 ± 1.96^a^	32.17 ± 2.46^b^	42.09 ± 3.17^c^	47.81 ± 2.71^d^
**Vittra**	27.17 ± 2.69^a^	46.96 ± 2.54^b^	50.04 ± 3.99^b^	54.25 ± 3.14^c^
**Filtek**	37.55 ± 0.98^a^	56.42 ± 1.21^b^	59.37 ± 1.14^c^	61.08 ± 1.13^d^

Different superscript letters indicate statistically significant differences between the different F/P systems for each composite material (*P* < .05).

**Fig. 4:**
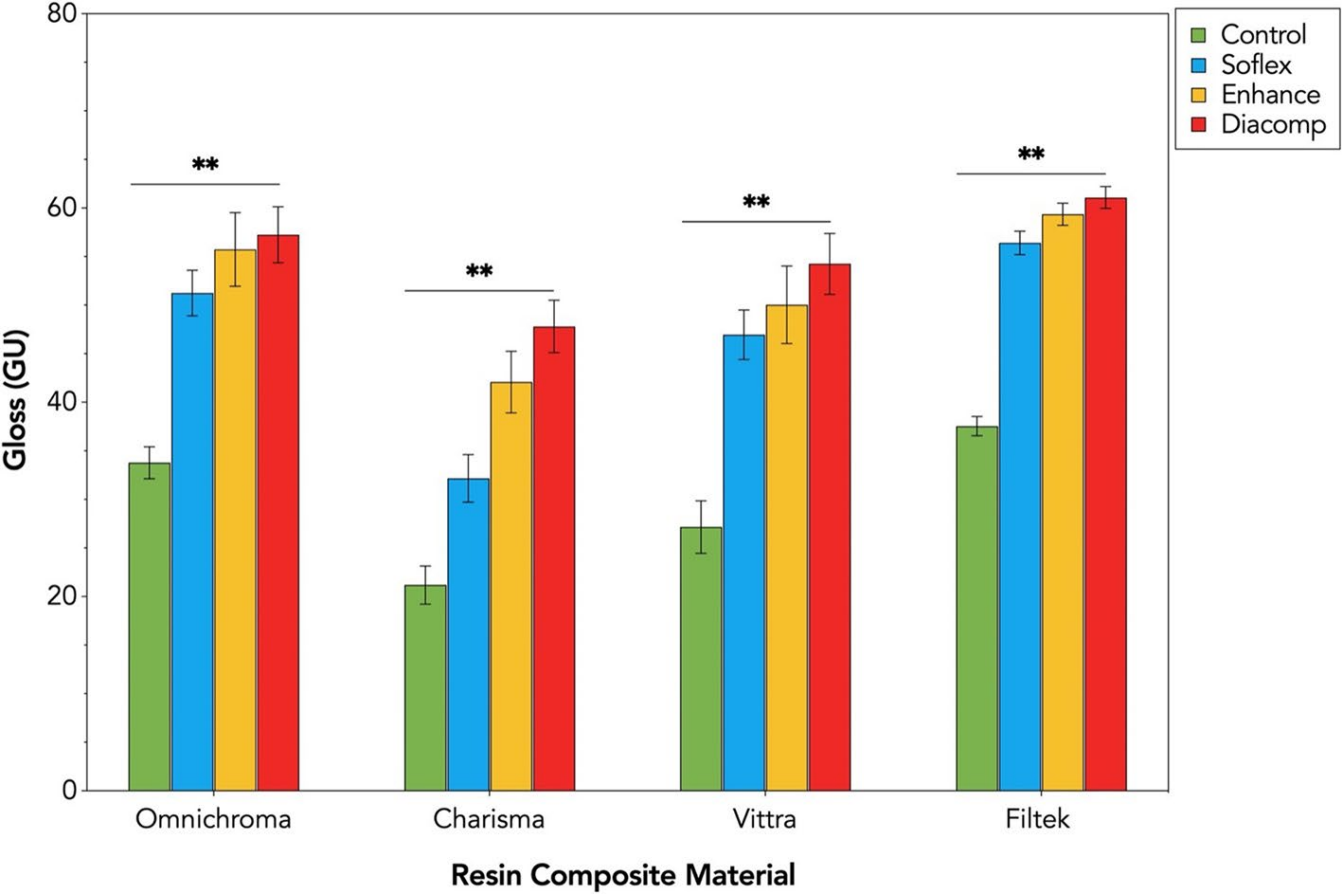
Gloss values (GU) of the tested resin composites based on the groups of finishing and polishing systems. Error bars represent ± SD. Double asterisks denote statistically significant differences at (*p* < .0001) among the different groups

Pairwise multiple comparisons with Tukey’s post hoc test showed Filtek had the highest GU values, which were significantly higher than all other tested materials (*p* < .000) (Fig. 4). The lowest GU values were observed for Charisma, which was significantly lower than all other tested materials (*p* < .000). In Omnichroma groups, no statistically significant difference was found between Enhance and Diacomp (*p* = 0.742), and in Vittra groups, no statistically significant difference was found between Soflex and Enhance (*p* = 0.146). The order of composites for all 4 F/P systems ranked from the highest to the lowest of GU was: Filtek > Omnichroma > Vittra > Charisma.

Regarding the F/P system, Tukey’s post hoc test showed that the use of Diacomp resulted in the highest GU of resin composites (*p* < .000) (Fig. 4). The lowest GU was observed after using Soflex (*p* < .000), but it was glossier than the Control (no finishing & polishing) group. The order by the F/P systems for all tested composites ranked from the highest to the lowest of GU was: Diacomp > Enhance > Soflex > Control.

### Vickers microhardness

The mean values and standard deviations of Vickers Microhardness (VMH) for each resin composite are presented in Table 5. The results indicated statistically significant differences (*p* < .000) and interaction effect between resin composite type and F/P systems, F (9,144) = 12,040.434, *p* = .000, partial Eta squared (*η*_p_^2^) = .849.

**Table 5:** Average Vickers Microhardness values (VMH) and standard deviations (± SD) of the tested resin composites for each group of the finishing and polishing systems

Resin Composite		Finishing and Polishing System		
	Control	Soflex	Enhance	Diacomp
**Omnichroma**	60.12 ± 0.73 ^a^	63.2 ± 0.45 ^b^	64.82 ± 0.52 ^C^	67.28 ± 0.60^d^
**Charisma**	71.57 ± 0.84 ^a^	82.16 ± 1.04 ^b^	82.68 ± 0.92^b^	85.89 ± 0.62 ^c^
**Vittra**	82.7 ± 0.49^a^	83.83 ± 0.44^b^	85.13 ± 0.41^c^	85.99 ± 0.57^d^
**Filtek**	125.02 ± 0.72^a^	128.59 ± 0.75^b^	130.15 ± 1.22^c^	130.89 ± 0.65^c^

Different superscript letters indicate statistically significant differences between the different F/P systems for each composite material (*P* < .05)

**Fig. 5:**
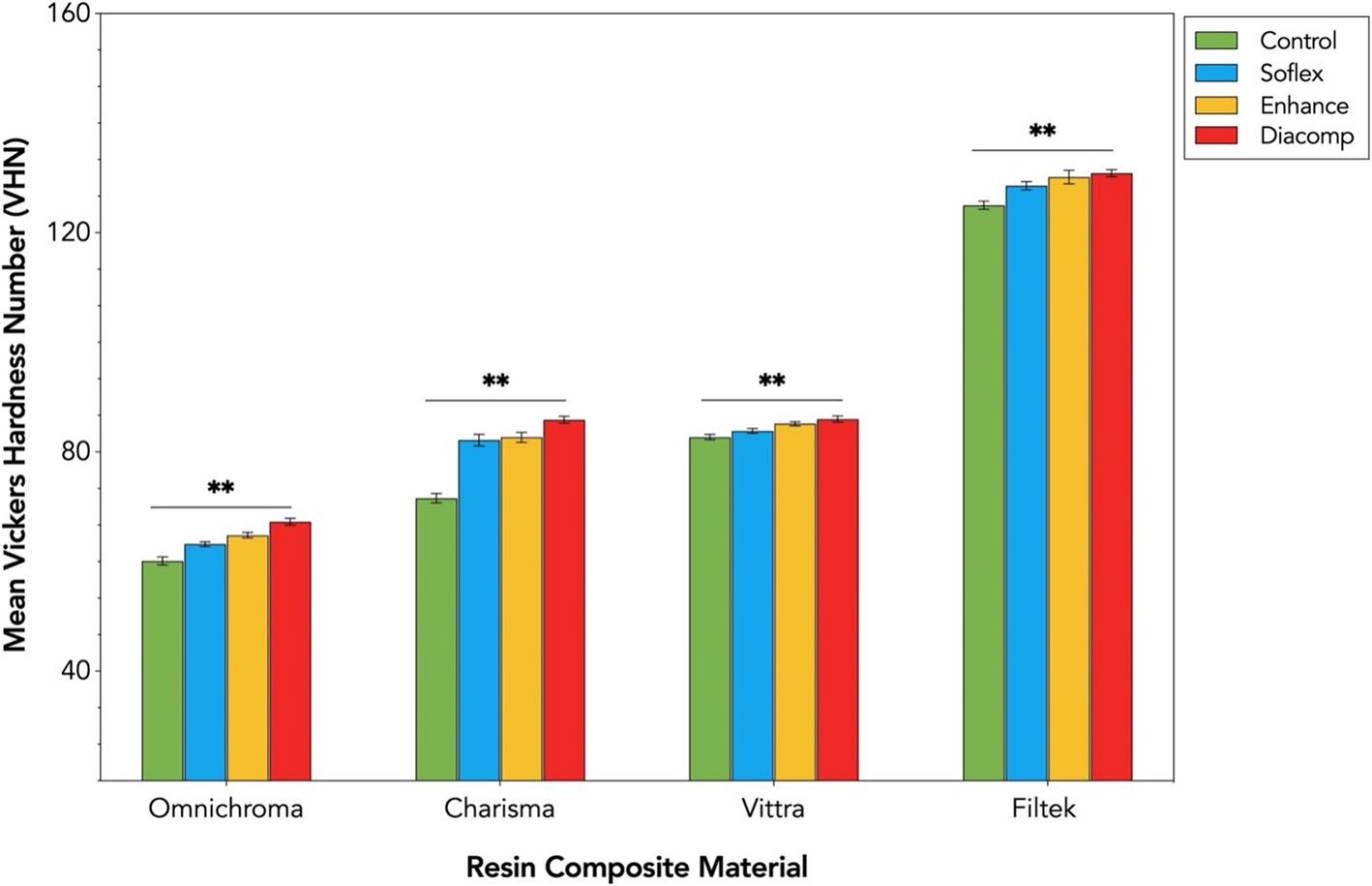
Mean Vickers Microhardness values (VMH) of the tested resin composites based on the groups of finishing and polishing systems. Error bars represent ± SD. Double asterisks denote statistically significant differences at (*p* < .0001) among the different groups

Pairwise multiple comparisons with Tukey’s post hoc test showed that Filtek has the hardest surface values than all other tested materials (*p* < .000) (Fig. 5). The lowest VMH values among the tested materials were observed for Omnichroma (*p* < .000). In Charisma groups, no statistically significant difference was found between Soflex and Enhance (*p* = 0.544), and in Filtek groups, no statistically significant difference was found between Enhance and Diacomp (*p* = 0.240). The order of composites for all 4 F/P systems ranked from the highest to the lowest VMH was: Filtek > Vittra > Charisma > Omnichroma.

Regarding the F/P system, Tukey’s post hoc test showed that the use of Diacomp resulted in the hardest surface of resin composites (*p* < .000) (Fig. 5). The lowest VMH values were observed after using Soflex (*p* < .000), but it was harder compared only to the Control (no finishing & polishing) group. The order of the F/P system for all tested composites ranked from the highest to the lowest of VMH was: Diacomp > Enhance > Soflex > Control.

### Correlation between Ra, GU & VMH

Spearman’s correlation showed a moderate positive correlation between the surface gloss and Vickers microhardness of the tested materials, which was statistically significant, (rs (158) = .414, *p* < .0001) (Table 6).

**Table 6:** Spearman’s Correlation matrix among properties of the tested resin composite materials

	Correlation Coefficient Spearman’s *rho (p-value)*		
	Surface microhardness (VMH)	Surface Roughness (Ra, μm)	Surface Gloss (GU)
Surface microhardness (VMH)	1		
Surface Roughness (Ra, μm)	−.190^a^ (*p* = 0.016)	1	−.855^b^ (*p* < .0001)
Surface Gloss (GU)	.414^b^ (*p* < .0001)		1

^a^ Correlation is significant at the 0.05 level (2-tailed)

^b^ Correlation is significant at the 0.01 level (2-tailed)

A weak negative correlation was found between surface roughness and Vickers microhardness, which was statistically significant (rs (158) = −.190, *p* < .016).

**Fig. 6:**
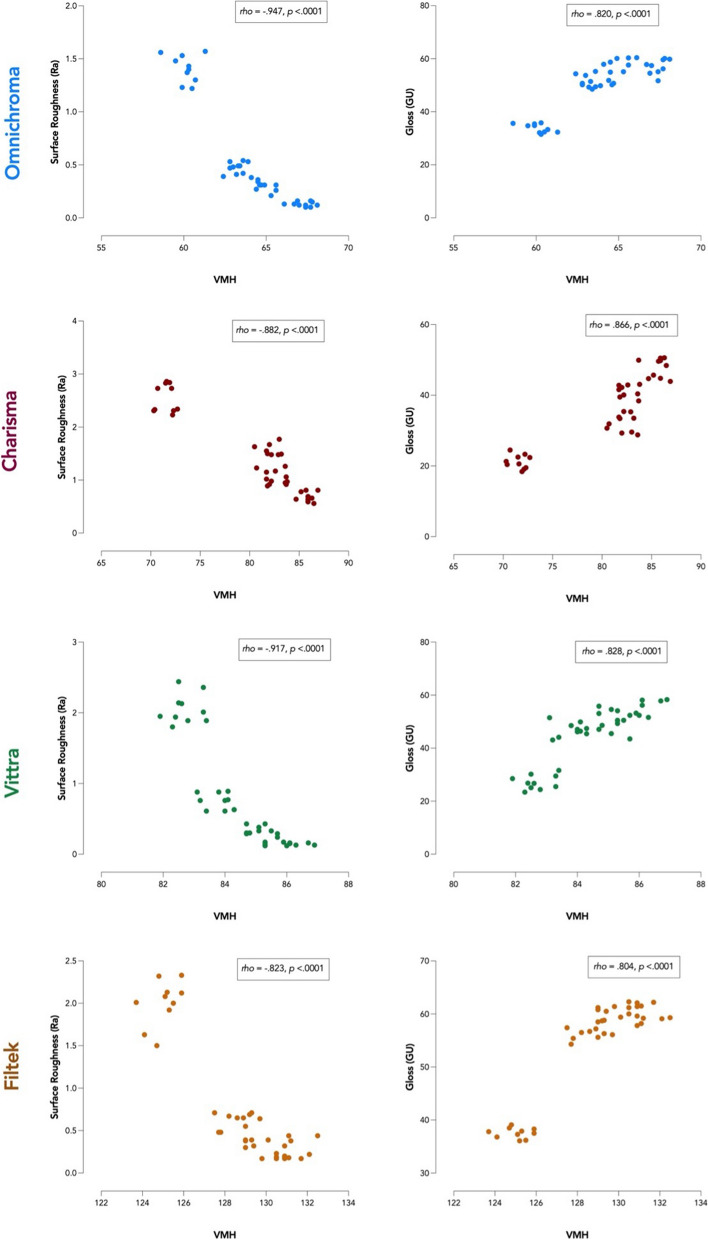
Spearman’s Correlations between Surface Roughness, Surface Gloss, and Vickers Microhardness among each group of the tested resin composite materials

A moderate negative correlation was found between surface roughness and surface gloss, which was statistically significant (rs (158) = −.855, *p* < .0001) (Fig. 6).

## Discussion

The surface properties are clinically relevant characteristics of restorative materials that should be considered to maintain the esthetic and functional requirements of the restored tooth and to predict the longevity of the restorations. This in-vitro study found that the two factors; finishing and polishing systems and resin-based composites had significantly influenced the surface roughness (Ra), surface gloss (GU), and Vickers microhardness (VMH). Hence, all four null hypotheses were rejected.

Several studies have shown that the effects of finishing and polishing procedures depend directly on both the restorative material and the F/P system [40–43]. The composite’s polishability is influenced by the resin matrix, as well as by filler type, size, and loading [44]. Relatively soft resin abrades faster than harder matrix, leading to surface irregularities [45]. It has also been reported that spherical particles allow for a better light reflection than irregular particles [40, 43, 46]. Surface quality is also greatly affected by the type and size of abrasive particles of the F/P systems applied [47]. The F/P abrasive type, size, and hardness are important factors that modify its effect on composite resin restoration. Speed and pressure of the F/P are important clinically, however, in this research these factors were standardized. The abrasive particles must be harder than the composite resin filler particles to avoid abrading only the resin matrix and leaving the filler particles protruding [48]. Also, the abrasive particles must be small to prevent deep scratches on the composite resin [49].

After the F/P procedures, the three tested F/P systems achieved better Vickers microhardness, surface gloss, and less surface roughness compared to the control groups where the surfaces were prepared with 800-grit silicon carbide (SiC) paper. For all the composite materials, Diacomp resulted in the smoothest surface of resin composites, while Soflex treated composites were the roughest. This is probably related to the type and particle size of abrasives used for F/P. Soflex and Enhance have aluminum oxide particles as the first step in finishing but the composition of the last polishing step was aluminum oxide for Soflex and Diamond-coated micro-polisher for Enhance, this difference in the composition may have explained how Enhance showed slightly better results than Soflex. The Diamond impregnated rubber spiral wheel resulted in the least surface roughness. This study is in accordance with previous studies, which found Diacomp groups yielded high surface microhardness values and low surface roughness values among the tested groups [50, 51]. Overall, Soflex and Enhance showed similar trends with minor differences between composite materials. This may be due to the ability of aluminum oxide-containing discs to produce a smooth surface which is thought to be related to their ability to cut the filler particles and matrix equally [52]. Similar results were reported by several authors [53–56]. In contrast to this study, Üçtaşli et al. [57] evaluated the effect of F/P systems on the surface roughness of a microfill, hybrid, and packable composite resin and concluded that Soflex discs produced a smoother surface than Enhance for all tested materials. Other reports corroborated these findings [58, 59]. Standardization of methodologies to investigate the effectiveness of F/P systems for RBCs could help to eliminate such conflicting findings.

Resin composites with smaller particle sizes have lower surface roughness and higher gloss after finishing and polishing [60]. Filler size reduction reduces particle projection at the surface which improves the surface roughness of the composite material. In the current study, Omnichroma had the lowest and Charisma had the highest surface roughness regardless of the F/P method employed. This could be due to the difference in the filler particle type, size, and shape. Omnichroma has unique uniformly sized supra-nano spherical filler and round-shaped composite filler (containing 260 nm spherical SiO2-ZrO2) which could have contributed to the smoothness of the restorations. On the other hand, Charisma has Barium Aluminium Boro Fluor Silicate with a larger particle size of 5 nm-20 μm. In addition, the pre-polymerized fillers can be gouged out due to their poor bond with the polymer matrix leaving large defects on the surface [61]. This could be seen as obvious circular depressions on the SEM images of Charisma composite with all F/P techniques [62]. More homogonous SEM images can be seen in Omnichroma because it is composed of super nanofilled composites. Similar to our results, Aytaç et al. [63] found that the lowest Ra value was observed in the supra-nano-filled composite resins compared to the other composite resin groups. Some studies reported contradictory results where no significant differences were found in surface roughness among different composites despite of their different filler sizes [60, 62, 64].

Chung found that restorations appear to be optically smooth when the Ra is lower than 1 μm [19]. The RBCs used in this study produced acceptable Ra values for all the tested F/P systems except the control (no finishing and polishing) and Charisma groups finished with Soflex and Enhance which exhibited Ra higher than 1 μm. This result can be explained by the composition of the material where Charisma is composed of a TCD-urethane-based matrix which enables a strong and rigid network that requires large and hard abrasive particles to be able to produce smooth surfaces [65]. Vittra and Filtek showed very similar patterns with regard to surface appearance, this finding could be related to their matrix composition or filler size. Vittra filler content was not disclosed by the manufacturer, however, according to the manufacturer Filtek is composed of surface-modified zirconia/silica with a median particle size of approximately 3 μm or less (non-agglomerated/non-aggregated 20 nm surface-modified silica particles). Furthermore, Filtek is characterized by compact spatial and molecular arrangement and fused nanocluster agglomerates which is believed to result in an improved surface smoothness [66, 67].

The surface gloss of restorations has been considered another important factor in the dental esthetics [68]. According to the ADA professional product review, 40–60 GU was identified as a typically desired gloss based on observations from an expert panelist [69]. In this study, gloss values achieved by all used F/P systems on all RBCs evaluated were greater than 40 GU except for the Charisma group polished with Soflex. This could be related to the larger filler size of Charisma which resulted in poor light reflection and lower gloss compared to the other tested material [61]. In addition to the characteristics of Charisma’s resin matrix that could mandate the sequential use of four different Soflex discs with longer application time to produce glossy surfaces with better light reflection [60].

Ra and GU are generally known to have a negative correlation, increased Ra accompanied by decreased GU. In the present study, this correlation can be seen clearly in Charisma groups. Similarly, some studies revealed a correlation between surface gloss and surface roughness [20, 70]. The results of this study did not consistently show that the improvement of surface roughness would lead to improvement of surface gloss. Also, the effect was material-dependent and most probably due to the interaction between RBCs filler size and the abrasive particles of F/P systems. Omnichroma groups showed the lowest Ra but not the highest GU, the glossiest surfaces were achieved by Filtek groups polished with Diacomp, Enhance, and Soflex. Previous studies reported that no relationship could be established between gloss and roughness [9, 53]. So, a smoother surface does not necessarily exhibit a high surface gloss, and the relationship depends on the F/P procedures and materials used. The higher gloss achieved by Filtek may indicate that conventional nanohybrid RBCs are better than the single-shade RBCs in terms of gloss by virtue of their optimized filler content, however, it is important to point out that most RBC materials & F/P systems combination were within the acceptable GU values except for Charisma.

Microhardness testing evaluates the resistance of a material to plastic deformation, usually by indentation, under a given load. Therefore, this property should be strongly related to the resin-based composite filler content, however, it is often difficult to explain differences found between commercial materials [2]. Surface hardness has been used to predict the wear resistance of a material and its ability to abrade or be abraded by opposing dental structures or materials [16]. Vickers microhardness values of dental composites spectrum range from 30 to over 100, however, to approximate the hardness of natural tooth tissues, the minimum VHN value is expected to be 40–50 [71]. Filtek had the highest surface microhardness values than all other tested materials, and the lowest VMH values among the tested materials were observed for Omnichroma. Filler loading affects the microhardness [2, 72], however, the two materials have similar filler content so the difference is again probably related to the filler type or resin component. Filtek contains Silica and Zirconia/Silica particles while Omnichroma has supra-nano spherical SiO2-ZrO2 fillers.

Effective finishing and polishing procedures are capable of reducing the surface roughness of composite materials and improving their surface gloss and surface microhardness [73]. Although the conventional nanohybrid RBC (Filtek Z250 XT) showed better GU and VMH values, the universal single-shade RBCs demonstrated comparable surface properties with the added advantage of simplified shade selection leading to less chair-time, as well as good esthetic and surface properties. This study found a moderate positive correlation between the GU and VMH, this is in agreement with multiple studies where a similar positive correlation between GU and VMH was found [12, 23]. A moderate correlation between Ra and GU [62, 74, 75] and a weak negative correlation between Ra and VMH were also found [23, 76, 77].

According to the literature [53, 78–80], the smoothest composite surfaces were obtained by well-placed mylar strips. However, in most cases, finishing the restoration is necessary to remove excess material and to contour the restoration. In addition, the polymerized surface against the matrix band is rich in the resin matrix and less resistant to abrasion, and it can contain air inclusion and folds [81]. Removal of the limiting resin layer, together with flash excess by finishing and polishing procedures tends to leave a harder, more wear-resistant, and esthetically stable surface [82]. In addition, finishing and polishing procedures are typically necessary to remove the oxygen-inhibited layer of resin composites. This results in a surface that is harder and more esthetically acceptable [83]. In case finishing and polishing procedures are difficult to achieve properly, the application of a light-curing protocol performed in the absence of oxygen may improve the chemicophysical properties, as well as the polishability of resin composites. The use of glycerin or argon gas may be suitable for light-curing procedures of occlusal surface in posterior teeth, while a mylar matrix can be used in esthetic areas [84].

This is an in-vitro study that does not replicate the intraoral conditions and it might present results that are different from the clinical situation. Some factors present in the oral cavity like saliva, enzymes, foods, and beverages with different pH levels and temperatures can affect the surface roughness, gloss, and microhardness of RBCs. Further in-vivo studies are necessary to evaluate the effect of F/P systems on the surface quality and longevity of the universal single-shade RBCs.

## Conclusions

Within the limitations of this study, the following conclusions can be made:

• Omnichroma showed the lowest Ra and an acceptable GU. On the other hand, it showed the lowest VMH, but it is still considered within the acceptable hardness value. So, it can be used to restore anterior teeth.

• Charisma showed the highest Ra, acceptable GU, and VMH. So, it is not recommended to be used in anterior teeth and can be used to restore posterior teeth.

• Vittra showed an acceptable Ra, GU, and VMH.

• Filtek showed the highest GU, VMH, and acceptable Ra.

• The highest GU & VMH and lowest Ra were achieved by Diacomp followed by Enhance and Soflex.

## Data Availability

All data generated or analyzed during this study are included in this published article. The raw data and results of statistical analysis are available upon request from the corresponding author.

## Abbreviations

RBCs: Resin-Based Composites
Ra: Surface Roughness
GU: Surface Gloss
VMH: Vickers Microhardness
F/P: Finishing & Polishing
OC: Omnichroma
CD: Charisma® Diamond one
VU: Vittra APS Unique
FT: Filtek™ Z250 XT
SX: Sof-Lex™ XT
EP: Enhance®/PoGo®
DT: Diacomp® Plus Twist
Bis-GMA: Bisphenol A diglycidildimethacrylate
UDMA: Urethane dimethacrylate
TEGDMA: Triethylene glycol dimethacrylate,
Bis-EMA: Bisphenol A ethoxylated dimethacrylate,
PEGDMA: Polyethylene glycol dimethacrylate,
HEMA: 2-hydroxyethyl methacrylate
TCD: Tricyclodecane
BHT: Butylhydroxytoluene.
LC: Light Cure
SEM: Scanning Electron Microscope
